# Midterm outcome after en bloc resection of C2 chordoma with transoral mandibular split and mesh cage reconstruction: a case report

**DOI:** 10.1186/s13256-023-03958-2

**Published:** 2023-06-03

**Authors:** Ekkapoj Korwutthikulrangsri, Sunun Ongard, Jirachai Pisutbenya, Monchai Ruangchainikom, Werasak Sutipornpalangkul

**Affiliations:** 1 Department of Orthopaedic Surgery, Faculty of Medicine Siriraj Hospital, Mahidol University, Bangkok, Thailand; 2 Department of Otorhinolaryngology, Faculty of Medicine Siriraj Hospital, Mahidol University, Bangkok, Thailand; 3 Division of Orthopedic Surgery, Golden Jubilee Medical Center, Mahidol University, Bangkok, Thailand

**Keywords:** Chordoma, En bloc resection, Fiberoptic endoscopic evaluation of swallowing, Mandibular split, Tracheostomy, Upper cervical spine

## Abstract

**Introduction:**

Chordomas are rare, locally aggressive tumors that often occur in the axial spine, especially in the sacrum. The treatment of chordomas located in the upper cervical spine is challenging. En bloc resection is the preferred surgical option for total tumor removal.

**Case presentation:**

We report the case of a C2 chordoma in a 47-year-old Thai woman. She was treated with a two-stage, anterior–posterior, C2 total spondylectomy with titanium mesh cage reconstruction and radiotherapy. The first stage involved posterior stabilization from the occiput to C5, a total laminectomy, and removal of the posterior rings of the bilateral foramen transversarium to preserve the bilateral vertebral arteries. The second stage comprised a transoral mandibular split with en bloc resection of C2, followed by titanium mesh cage reconstruction and kick-off anterior cervical plating. At the 5 year follow-up, no tumor recurrence was identified on magnetic resonance imaging. The patient had no neurological deficits but still had minor complications from the anterior transoral mandibular split.

**Conclusions:**

Excellent midterm results were obtained using a transoral mandibular split with reconstruction and posterior spinal fusion from the occiput to the lower cervical spine coupled with adjuvant radiotherapy. We recommend this approach as the treatment of choice for chordoma in the upper cervical spine.

## Introduction

Chordomas are exceedingly rare, slow-growing tumors with an overall incidence of 8.4 per million patients. With their high morbidity and mortality rates, they have a poor prognosis. The median survival time is only 6.29 years due to their high local aggressiveness [1]. Surgical resection is the primary treatment, typically followed by radiotherapy. However, complete tumor resections are particularly challenging in some areas. This is especially the case with the upper cervical spine because of its unique anatomical structure and intimate relationships with the vertebral arteries, cervical nerve root, and spinal cord [2, 3].

Chordomas originate from the remnants of embryonal notochords. These bone cancers are described as midline lesions involving the skull base or sacrum and, less frequently, the cervical spine. Because chordomas are considered relatively radioresistant and locally aggressive, en bloc resection is recommended. The procedure is associated with prolonged recurrence-free survival and is a potential cure for some patients [4, 5]. However, en bloc resection of chordomas in the upper cervical spine is challenging. This is because of the unique anatomy of that region and its proximity to the surrounding vital structures (vertebral arteries, cervical nerve roots, and spinal cord). Many authors have described en bloc resection of C2 chordomas [5–13]. Most articles are case reports with literature reviews (Table 1). Moreover, most methods involve two-stage surgery that utilizes anterior and posterior techniques to optimize spinal stability and achieve complete tumor removal. In addition, an extended transoral transmandibular approach was employed, and only short-term outcomes were reported. Therefore, our group reports a chordoma patient’s midterm (5 year) outcomes and complications. The individual underwent a posterior approach for fixation of the cervical spine and preservation of the bilateral vertebral arteries. Anterior en bloc resection of the C2 chordoma was performed using only the transoral mandibular split approach.

**Table 1:** Literature review of previous studies on en bloc resection of C2 chordoma

Paper	Year of publication	Age (years)/sex	Type of surgical approach	Type of implants	Duration of follow-up	Outcomes
Muhlbauer *et al*. [6]	2001	54/M	Lateral transfacetal retrovascular approach	Iliac crest bone graft	6 months	Fully ambulatory with no neurological deficit
Suchomel *et al*. [9]	2013	62/M	Transoral and posterior approach	Harms mesh cage	17 days	No neurodeficit except C2 distribution hypesthesia
Ortega-Porcayo *et al*. [5]	2014	43/M 23/F	Extended transoral transmandibular approach and posterior approach	1. Titanium cage 2. Nonvascularized autologous fibular graft	2 years	Improved neurological without hardware failure
Taran *et al*. [10]	2015	44/F	Transoral endoscopic resection and posterior fixation		3 years	Free from recurrence and no neurological deficit
Weil *et al*. [12]	2015	46/F	Transoral transmandibular	Titanium mesh cage	40 months	Functioning normally, no deficit in speech and swallowing
Ozpinar *et al*. [7]	2016	48/F	Midline labiomandibular glossotomy	Expandable cage	26 months	Disease free without neurologic deficit
Wewel *et al*. [13]	2016	35/M	Transoral and posterior approach	Expandable cage	6 months	Intact neurological status without any significant morbidity
Tenny *et al*. [11]	2017	55/F	Anterior transmandibular and posterior approach	Pyrimesh cage	10 months	Tumor free
Petrov *et al*. [8]	2019	27/M	Transoral robotic surgery with a posterior approach	Iliac crest bone graft	15 months	Disease free
The present study		47/F	Transoral transmandibular and posterior approach	Titanium mesh cage	5 years	No recurrence without neurological deficit

## Case presentation

**Fig. 1:**
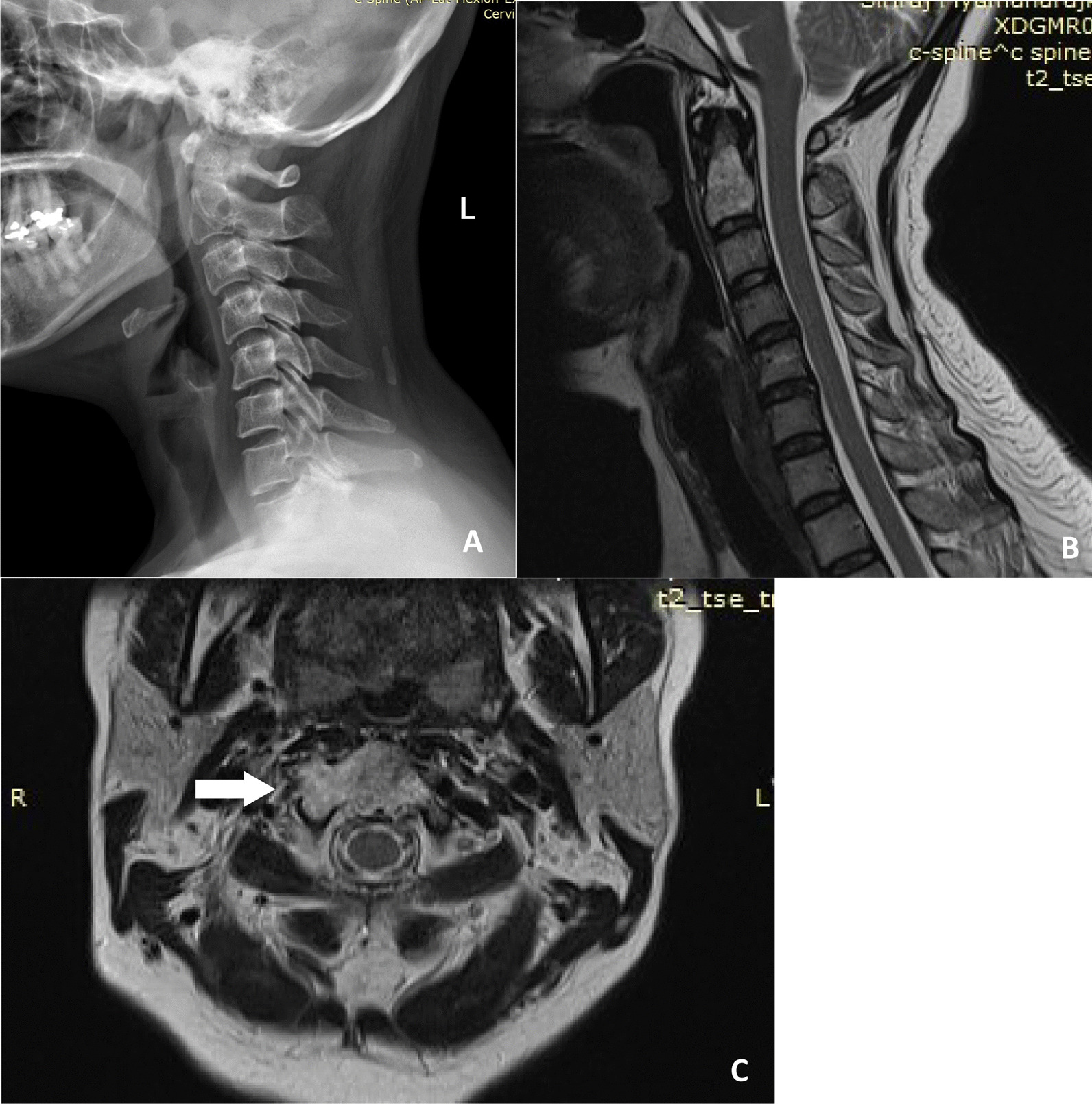
Preoperative plain cervical spine radiograph showing loss of cervical lordosis and mild narrowing at the C5–6 intervertebral discs (**A**) and T2-weighted magnetic resonance images (sagittal section, **B**; axial section, **C**). The magnetic resonance imaging revealed a heterogeneous mass involving C2, with an extension to the right foramen transversarium (white arrow)

**Fig. 2:**
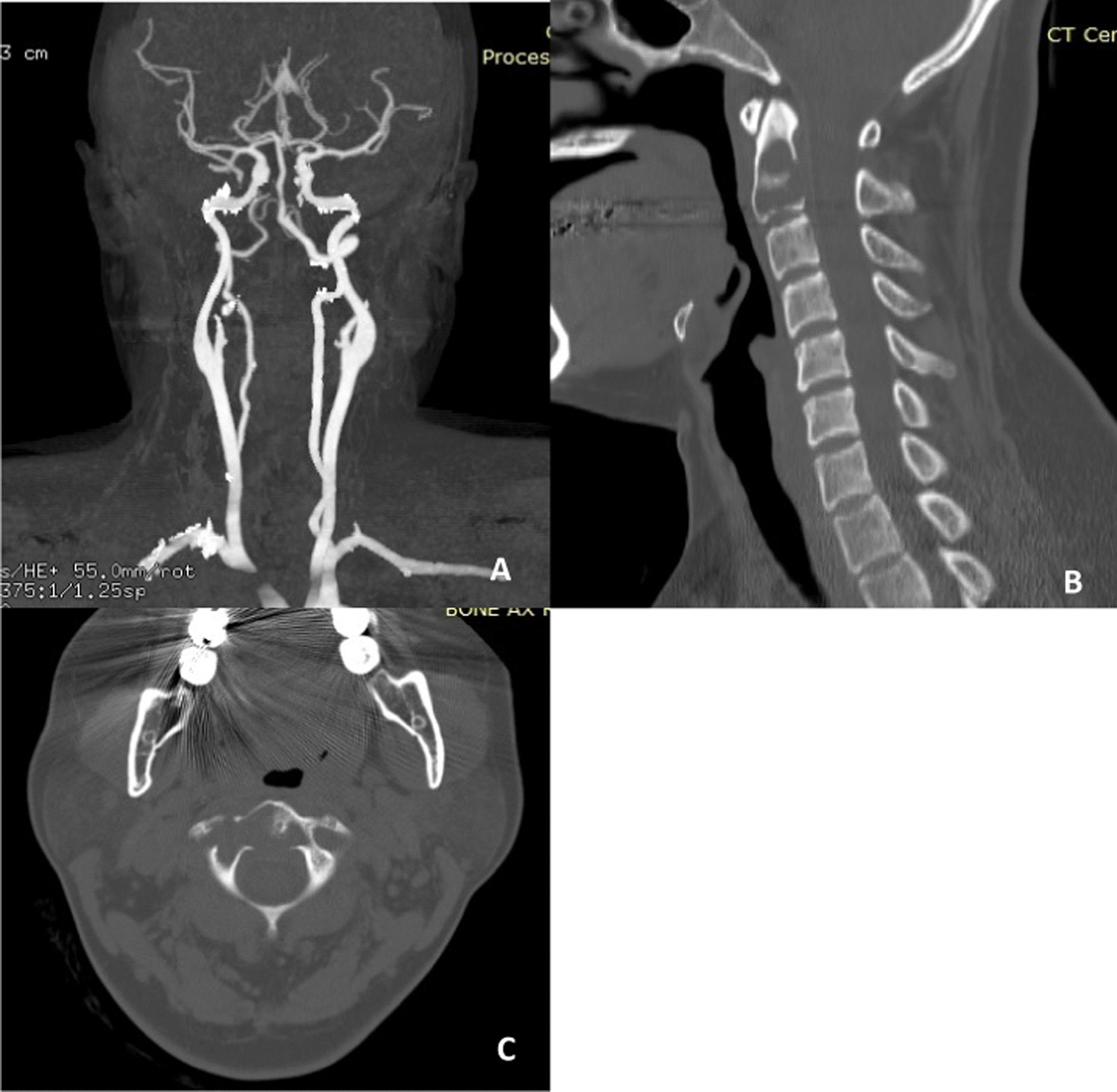
Computed tomography angiography (**A**) revealed no abnormalities of the bilateral vertebral arteries and a right-side prominence. Sagittal (**B**) and axial (**C**) computed tomography of the cervical spine showed a lytic lesion at the body of C2 without sclerotic calcification

A 47-year-old Thai female presented with mild neck pain radiating to the left arm and numbness in her left hand for 3 months. She denied any significant medical history, and a physical examination was unremarkable. Plain radiography of her cervical spine only showed a loss of cervical lordosis and mild narrowing at the C5–C6 foramen (Fig. 1A). Her symptoms did not improve after 2 weeks of conservative treatment, so magnetic resonance imaging (MRI) of the cervical spine was performed. It revealed a C2 vertebral-body tumor (Fig. 1B, C). We reviewed her plan of treatment in consultation with her primary orthopedic doctor. It was decided to perform a tumor biopsy via a posterior approach at a private hospital. The subsequent pathological analysis reported a chordoma of the C2 cervical spine. The patient was referred to Siriraj Hospital, a tertiary-care center for surgical resections. We arranged for computed tomography angiography of her cervical spine to evaluate her bilateral vertebral arteries (Fig. 2A) and to obtain data for our spine navigation system (Fig. 2B, C). After consultation with an ear, nose, and throat surgeon, we undertook a two-stage procedure. The first stage involved posterior instrumentation fusion with preservation of the bilateral vertebral arteries. One week later, the second stage was conducted. It comprised a transoral mandibular split, titanium mesh cage reconstruction, and kick-off anterior cervical plating.

## Operative details

For the first stage of surgery, the patient was transferred to the operative room, intubated, and placed in the prone position with three-point Mayfield-clamp head fixation. A neuromonitoring system was applied and checked for reliability. Xylocaine with adrenaline was administered at the surgical site to decrease bleeding during surgical dissection. The dissection was performed from the occiput to C5. An occiput plate (Vertex Max; Medtronic Inc., Minneapolis, MN, USA) was applied to the posterior of the occiput. An O-arm navigation reference array was placed on spinous process C2. Bilateral pedicle screws (Vertex, Medtronic Inc.) were applied from C3 to C5 with a surgical navigation system (StealthStation; Medtronic Inc.). After that, a C2 total laminectomy was performed. This was followed by identifying the posterior rings of the bilateral foramen transversarium and its subsequent removal with a high-speed burr drill until the bilateral vertebral arteries were free. We carefully freed the bilateral vertebral arteries from the foramen transversarium to prevent their injury during anterior removal of the C2 vertebra in the second surgical stage. Appropriate rods were fitted between the occiput plate and the C3–C5 pedicle screws, with iliac bone grafts placed laterally to enable spinal fusion. The wound was closed in layers with a Jackson–Pratt surgical drain left inside. No notable changes were observed in the neuromonitoring system, and the patient woke without any neurological deficits. Postoperatively, computed tomography angiography was repeated. The total resection of the bilateral posterior arch of the C2 foramen transversarium was confirmed. The patient remained in the hospital for 1 week before the next stage was performed.

**Fig. 3:**
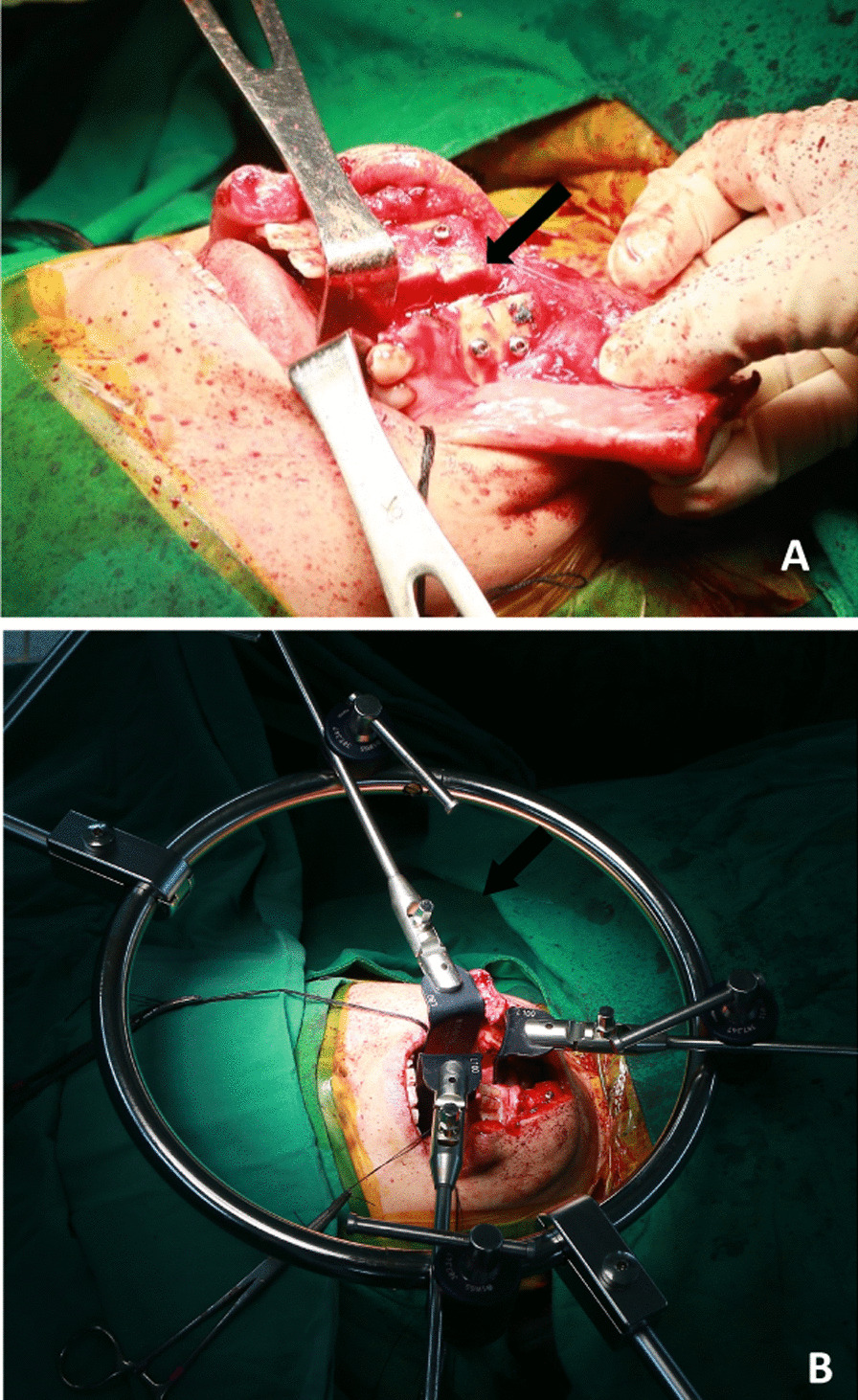
Intraoperative picture showing the zigzag splitting of the mandible (**A**) (black arrow) and surgical exposure after application of the table-mounted retractor system (**B**)

For the second stage of surgery, the patient was returned to the operating theater, intubated, and placed supine on the operating table. Ventilation via tracheostomy was chosen. Doing so not only secured a satisfactory view of the surgical field but also ensured comfortable patient airway handling, thereby preventing postoperative airway obstruction. An ear, nose, and throat surgeon performed a left paramedian mandibulotomy via a midline lip-splitting incision. A midline palatal split followed this to increase the surgical exposure field at the nasopharyngeal level. A linear incision was made from the lower eyelid to the chin, and then dissection from the skin to the mandibular bone was performed. A reconstruction plate was placed at the anterior mandible for final wound closure, but the plate was removed to perform a mandibulotomy. Tooth number 33 was extracted. The mandibular bone was then split between teeth numbers 32 and 34 using an oscillating saw in a zigzag manner (Fig. 3A). The soft palate was split in the midline. A table-mounted retractor system (SynFrame; DePuy Synthes, West Chester, PA, USA) was applied transorally. The retractor pushed the posterior tongue into a forward and lower position while pushing both sides of the buccal mucosa laterally. The positioning of the tongue was checked by intraoperative fluoroscopy to ensure adequate exposure from C1 to C3 with no limitation of the surgical field (Fig. 3B). The retractor was released intermittently to prevent tongue ischemia. The anterior arch of C1 was removed with a high-speed burr drill, thereby releasing the atlantooccipital ligament. Dissection of the body of C2 was carried out. This involved lateralization to the bilateral foramen transversarium and identification of the bilateral vertebral arteries. Complete discectomy and posterior longitudinal ligament removal between C2 and C3 were performed. Finally, en bloc resection of the body of C2 was carried out, and a specimen was submitted for pathological analysis.

For the reconstruction, a titanium mesh cage (Pyramesh; Medtronic Inc.) was cut into a T-shape on one side. A bone graft harvested from the iliac crest was used to fill the cage. With the aid of fluoroscopy, minicortical screws were used to attach the cage bilaterally to the C1 lateral masses. A T-shaped superior-aspect fixation was employed. The inferior aspect of the cage was secured using anterior cervical plating as the kick-off plate. During plating, Ethibond sutures (size 2; Ethicon Inc., Somerville, NJ, USA) were wrapped over the inferior aspect of the cage to the cervical plate to prevent posterior migration of the cage into the spinal canal. The surgical area was irrigated with normal saline solution. Intraoperative 3D imaging (O-arm; Medtronic Navigation, Louisville, CO, USA) was obtained to confirm the proper positioning of the cage and cervical plate. The split mandible was reduced and fixed with the previous fixation reconstruction plate. The retropharyngeal tissue and soft palate were closed with absorbable sutures, while the skin was closed with subcutaneous sutures.

## Postoperative treatment

**Fig. 4:**
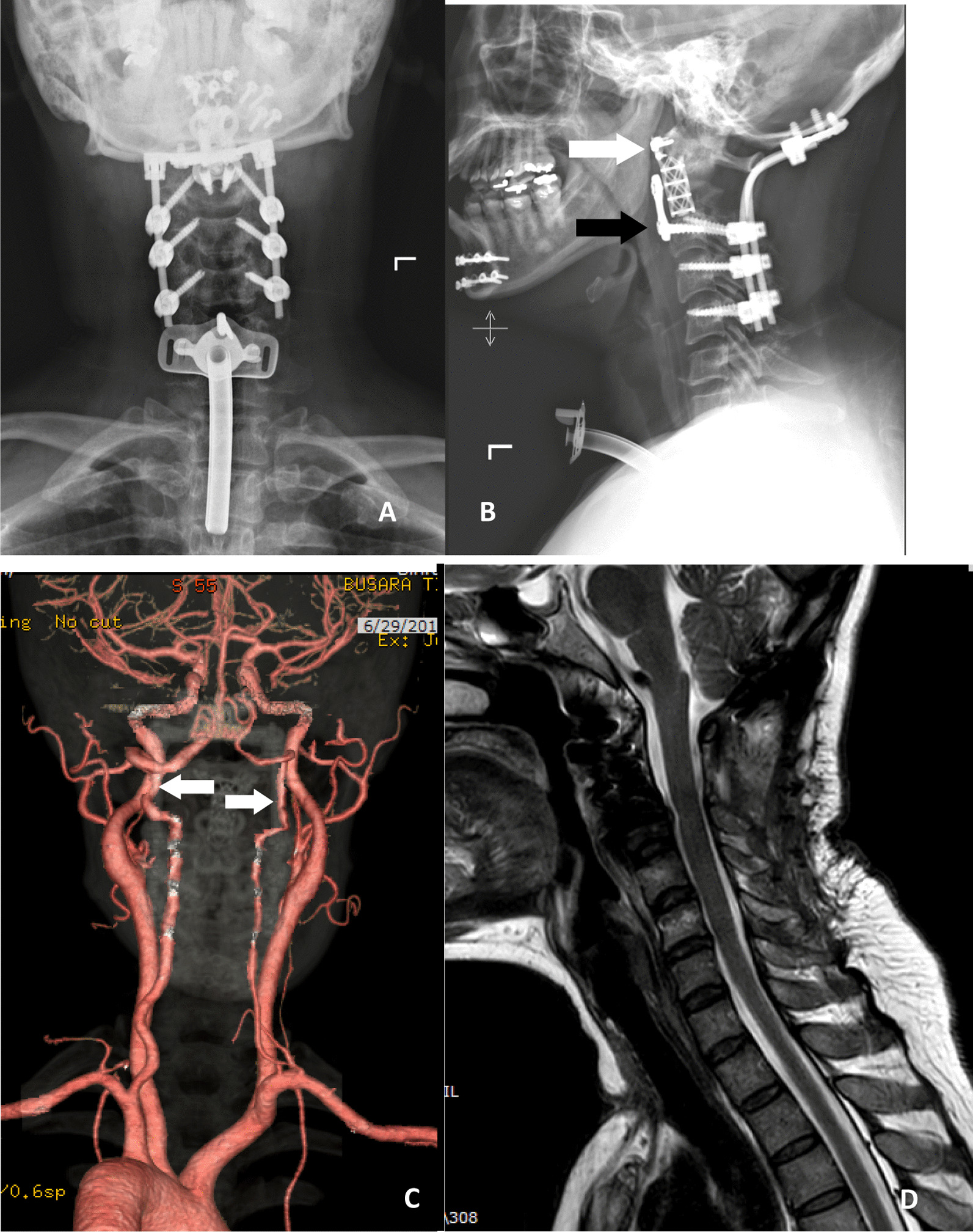
An immediate postoperative plain radiograph of the cervical spine showing the titanium mesh cage (white arrow) with the anterior kick-off plate (black arrow) and posterior instrumentation from the occiput to C5 (**A**, **B**). Patency of the bilateral vertebral arteries was apparent upon computed tomography angiography (white arrow) (**C**). A T2-weighted sagittal cervical spine MRI revealed no tumor mass and demonstrated an intact spinal cord (**D**)

Plain radiography was performed immediately after surgery (Fig. 4A, B). The patency of the bilateral vertebral arteries was also confirmed by computed tomography angiography (Fig. 4C). A sagittal T2-weighted cervical spine MRI showed no abnormal signal intensity and demonstrated an intact spinal cord without spinal injury (Fig. 4D). The surgical drain was removed on postoperative day 5. An ear, nose, and throat team managed the tracheostomy care and feeding route. The tracheostomy was retained with a plastic, cuffed tracheostomy tube (Shiley; size 6; Medtronic Inc.). On postoperative day 1, tube cuff inflation of 6 ml of air was used to prevent blood leakage from the tracheostomy wound entering the trachea. On postoperative days 2 and 3, the pressure was released to avoid damage to the subglottis. On postoperative day 4, the plastic tracheostomy tube was replaced by a silver tube (still size 6). This improved the patient’s comfort because silver tubes are less irritating and easier to manage than plastic tubes. Since this patient did not need further surgery, decannulation was planned by stepping down from tube size 6 to size 4. The tracheostomy tube was occluded during the daytime, with clinical observation for dyspnea. As the occlusion was uneventful, day and night occlusion was applied. The tracheostomy tube was removed on postoperative day 22, and the skin wound was closed with a petrolatum gauze pressure dressing. A fiberoptic endoscopic evaluation of swallowing was conducted. The oral preparation was normal in the oral phase. No bolus retention, penetration, or aspiration was evident in the pharyngeal phase. However, there was minimal regurgitation with retained secretions plus remnant food at the surgical wound. Given that the patient could eat orally, her nasogastric tube was removed, and nasal irrigation after meals was advised. The patient had no neurological deficits after surgery. She began radiation therapy due to an extraosseous extension of the chordoma. A Philadelphia collar immobilized her neck for 6 weeks after surgery.

**Fig. 5:**
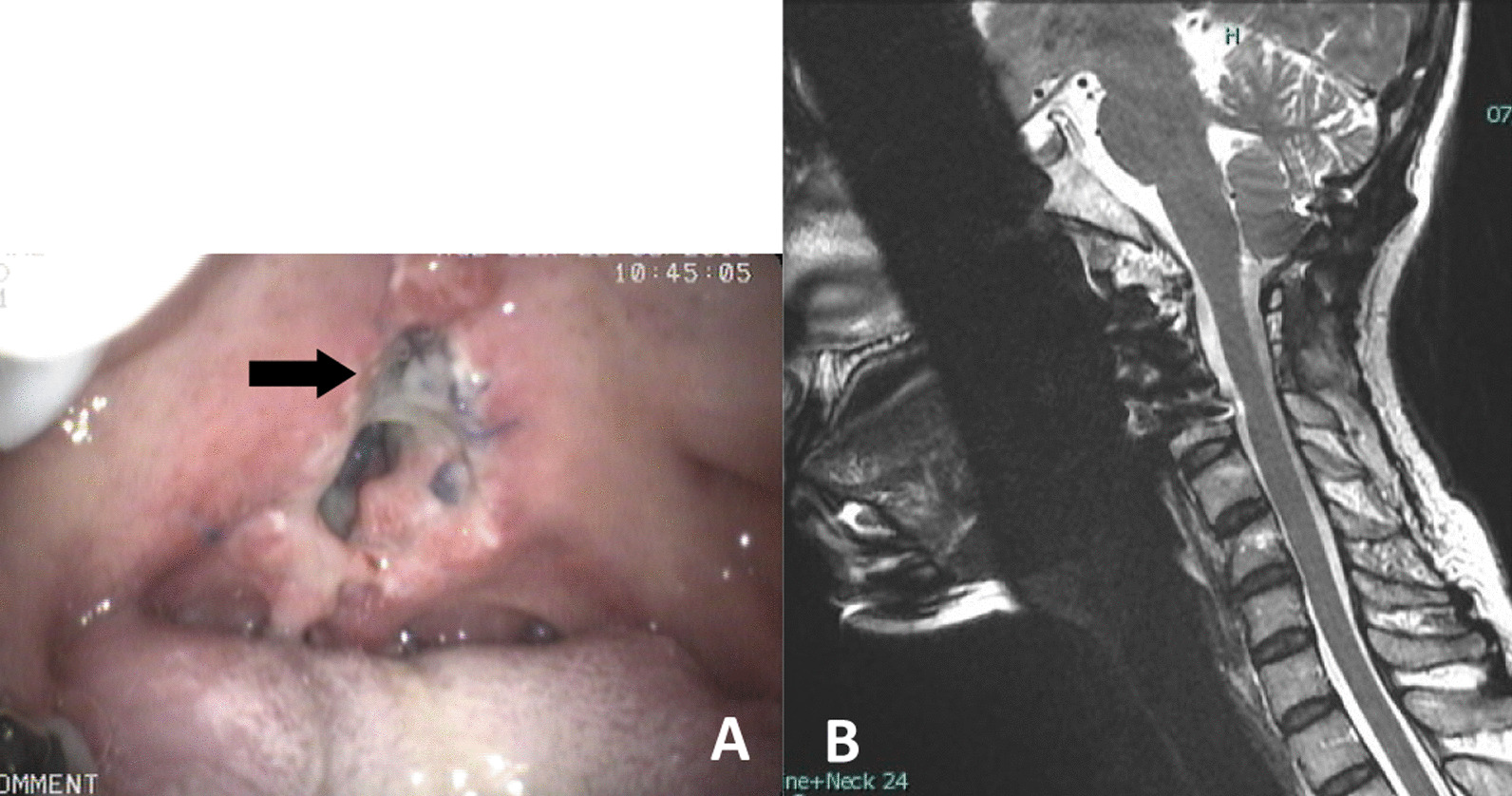
Surgical wound dehiscence and the exposed anterior cervical plate (black arrow) were observed before radiotherapy (**A**). Midterm (5 year) follow-up magnetic resonance imaging of the cervical spine revealed no gross tumor recurrence at the surgical bed (**B**)

During the follow-up period before the commencement of radiotherapy, there was wound dehiscence and granulation tissue formation at the nasopharynx surgical wound. The patient also developed a swelling mass in her throat that caused dysphagia and changed her voice. The lesion was therefore investigated by endoscopy. After it was excised for pathological examination, the base of the mass was inspected (Fig. 5A). Wound dehiscence with exposure of the anterior cervical plate was found. Resuturing of the wound was performed at the posterior pharyngeal wall. The sutures ran from the lower half of the nasopharynx to the upper half of the oropharynx; they were placed under the pharyngeal mucosa and the approximated mucosa. The suturing used a minimal tension force and four 2–0 Vicryl stitches. The pathology of the lesion was reported as granulation tissue without recurrence of the chordoma tumor. Finally, adjuvant radiotherapy was applied to the surgical bed to achieve microscopic tumor destruction.

At the 5 year follow-up, rigid spinal fusion at the anterior and posterior reconstruction was observed without apparent loosening of the spinal implant. The midterm follow-up MRI of the cervical spine also revealed no gross recurrence of the tumor (Fig. 5B). The patient still had some minor complications from anterior transoral mandibular split such as mild dysphagia, throat discomfort after meal, and hypernasal voice,but they did not affect her function.

## Discussion

Surgical treatment of chordomas of the upper cervical spine is technically demanding due to the anatomical complexity of the region and its proximity to the vertebral arteries, cervical nerve root, and spinal cord. En bloc resection of a tumor with free margins was found to have an excellent long-term prognosis and survival [4, 5]. However, most studies still use postoperative radiotherapy as an adjuvant treatment [13]. With our patient, radiotherapy was utilized to destroy the microscopic remnants of the tumor due to the extraosseous extension of the tumor into the right foramen transversarium. At the midterm (5 year) follow-up of our patient, no recurrence of the tumor was observed in an MRI scan of the cervical spine. Therefore, surgical treatment and adjuvant radiotherapy are recommended for patients with chordoma in the upper cervical spine.

Various spinal stabilization and reconstruction techniques after resectioning a chordoma in the upper cervical spine have been reported [5–13]. Spinal fusion from the occiput to the lower cervical spine is the most preferred technique for posterior stabilization. However, the method for anterior reconstruction varies, depending on surgeons’ preferences. For example, fibular grafts, titanium mesh cages, expandable cages, and three-dimensional printed cages are employed. Our group used a titanium mesh cage filled with an iliac bone graft. The cage was applied after a transoral mandibular split. There was no need for extended surgical exposure, which decreased the likelihood of morbidity-related complications developing. We also modified the fixation of the T-shaped upper end of the titanium mesh cage by using minicortical screws to attach the cage bilaterally to the C1 lateral masses. Moreover, we wrapped Ethibond over the lower end of the cage and employed anterior cervical plating as the kick-off plate. Our modified anterior fixation system provided an excellent level of stability comparable with that achieved with the fixation techniques described by Tenny *et al*. [11] and Weil and colleagues [12]. The presence of rigid spinal fusion and the absence of implant loosening at the midterm follow-up confirmed the success of our approach.

## Conclusions

An anterior transoral mandibular split with titanium mesh reconstruction proved effective when combined with posterior spinal fusion from the occiput to the lower cervical spine. Given the excellent midterm outcomes, this approach is recommended as a treatment for chordomas of the upper cervical spine.

## Data Availability

All data, including the patient’s informed consent, are included in this published article.

## Abbreviation

MRI: Magnetic resonance imaging
